# The Early Diagnosis of Gas Gangrene

**Published:** 1919

**Authors:** 


***The Early Diagnosis of Gas Gangrene.*** By FREDERICK CHRISTOPHER, Lieut. M. R. C. Preliminary notes on an article to appear elsewhere.

The author emphasizes the great importance of early diagnosis in case of gas gangrene. He cites as evidence of this need the statistics of deaths in the American hospitals in Paris during a recent drive. Thirty-three per cent. were due chiefly to gas gangrene. In most cases the diagnosis of the condition had been made too late.

The author confines his attention exclusively to the condition of "acute gaseous distention" which succeeds the first or "dormant stage" of the infection; first, because of the difficulty of diagnosis in this stage; and second, because proper surgical treatment at this point in the development of the infection is usually effective in checking its progress.

The following are the points to consider in diagnosis of this stage:

1. Pain in region of the wound.

2. Age of wound. Gas infection should be suspected in all wounds, especially a wound by shell fragments, for a period of at least six or eight days.

3. Mental state clear.

4. Temperature not a striking symptom — sometimes slightly raised, sometimes lowered.

5. Pulse is frequently increased, but this symptom is unaccompanied by a corresponding rise in temperature. This inconsistency is frequently suspicious. Crossing of the pulse and temperature curves Blake considers very characteristic.

6. Dyspnea has only prognostic significance.

7. Dry tongue, usual. Vomiting not very common.

8. Location. Gas gangrene is essentially a disease of the muscle, as Taylor has shown, especially of large muscle masses, such as thigh, shoulder, and calf.

9. Nature of wound. Shell fragments buried in tissues are commonly the centers of infection, and even through-and-through bullet wounds with sealed entrances and exits. Wounds that have not been surgically cleansed are especially prone to infection because of the pressure of traumatized tissues which are the favorable medium for the development of the gas bacillus and other agents.

10. The drainage of gas by surgical means is important, for, if confined, its mechanical action is a probable means of the spread of the infection. The action of the confined gas, as well as injury to important vessels, may serve to devitalize tissue and render it a ready medium for the development of infection.

11. Part subjected to pressure, like buttocks and back, should be held in suspicion of metastatic gas infection.

Swelling is the most important single sign. Any swelling about the wound should be suspected. Swelling is usually fusiform, as gas follows muscle bundles. A large swelling is unquestionably gaseous.

Color of skin is of great importance. First the skin becomes pallid because of pressure, then a dirty cream tint gradually appears, and may be taken as a sure sign that gangrene is established. Then regular purple areas appear, which enlarge and coalesce. Blebs form at this time and the dermis under them is a shining purple red. Finally a yellowish green tint is seen.

Color changes in the muscles are to a brick red, green, and finally black. Discharge is marked by an absence of suppuration. If it is increased, thin, and of a pale brown color, gas infection is to be suspected. It varies in color from red-brown to violet.

Blebs are too late a development to help in early diagnosis. Odor from the wound is exceedingly characteristic. It may be described as fetid, penetrating, resembling that from the thin stool of an infant suffering from extreme arteritis and acidosis. Taylor recognizes in it the odor of butyric acid.

Crepitus upon palpation has long been recognized as an accompanying symptom. It is due to the infiltration of the gas. The muscles become resonant from gas before they are crepitant to the finger. It is, at any rate, a comparatively late symptom, and may possibly occur when gas infection is not involved, as from serum injection, mechanical emphysema, gas hematoma, phlegmon, etc.

Elasticity on palpation is notable. Induration is not uncommon. Tenderness upon palpation is general, but not acute pain except when the infection is mixed.

There may be edema (due to B. œdematiens, Weinberg and Séguin, or to venous obstruction).

Muscles which seem to be dead upon pinching or bleeding should be suspected of harboring infection.

Percussion yields a tympanic note, if the limb is distended with gas. This is an important early sign. Auscultation with stethoscope aids in early detection of crepitus.

X-ray examination helps to establish the presence of the gas. It is, however, liable to confusion, as the appearance of gas bubbles may also result from other causes, such as enclymosis, syringing, etc.

Culture of the wound is too slow a process to help in early diagnosis. A fatal condition may be reached before laboratory returns are available.

Diagnostic aspiration of gas from wound may be helpful in diagnosis. Microscopic examination of smears is of little value. Acid reaction of wound (Almroth Wright) has but slight clinical value.

